# Cross-cultural adaptation and psychometric testing of the Dutch and German versions of the Evaluation of Daily Activity Questionnaire in people with rheumatoid arthritis

**DOI:** 10.1007/s00296-020-04657-7

**Published:** 2020-07-26

**Authors:** Alison Hammond, Jorit Meesters, Karin Niedermann, Alan Tennant, Thea Vliet Vlieland, Sarah Tyson, Ulla Nordenskiöld

**Affiliations:** 1 Centre for Health Sciences Research, Allerton, University of Salford, Frederick Road, Salford, M6 6PU UK; 2 Department of Orthopaedics, Rehabilitation and Physical Therapy, Leiden University Medical Center, Albinusdreef 2, 2333 ZA Leiden, The Netherlands; 3 Institute of Physiotherapy, Zurich University of Applied Sciences (ZHAW), School of Health Professions, Technikumstr. 71, 8401 Winterthur, Switzerland; 4 Leeds Institute of Rheumatic and Musculoskeletal Medicine, University of Leeds, Leeds, UK; 5 Department of Orthopaedics, Rehabilitation and Physical Therapy, Leiden University Medical Center, Albinusdreef 2, 2333 ZA Leiden, The Netherlands; 6 Division of Nursing, Midwifery and Social Work, University of Manchester, Manchester, UK; 7 Department of Clinical Neuroscience and Rehabilitation, University of Gothenburg, Gothenburg, Sweden

**Keywords:** Rehabilitation, Outcome assessment, Activities of daily living, Rheumatoid arthritis, Rasch analysis, Occupational therapy

## Abstract

The Evaluation of Daily Activity Questionnaire (EDAQ) is a detailed patient-reported outcome measure of activity ability. The objective of this research was to assess the linguistic and cross-cultural validity and psychometric properties of the EDAQ in rheumatoid arthritis for Dutch and German speakers. The EDAQ was translated into Dutch and German using standard methods. A total of 415 participants (Dutch *n* = 252; German *n* = 163) completed two questionnaires about four weeks apart. The first included the EDAQ, Health Assessment Questionnaire (HAQ) and 36-item Short-Form v2 (SF-36v2) and the second, the EDAQ only. We examined construct validity using Rasch analysis for the two components (Self-Care and Mobility) of the Dutch and German EDAQ. Language invariance was also tested from the English version. We examined internal consistency, concurrent and discriminant validity and test–retest reliability in the 14 EDAQ domains. The Self-Care and Mobility components satisfied Rasch model requirements for fit, unidimensionality and invariance by language. Internal consistency for all 14 domains was mostly good to excellent (Cronbach’s alpha ≥ 0.80). Concurrent validity was mostly strong: HAQ *r*_s_ = 0.65–0.87; SF36v2 *r*_s_ = − 0.61 to − 0.87. Test–retest reliability was excellent [ICC (2,1) = 0.77–0.97]. The EDAQ has good reliability and validity in both languages. The Dutch and German versions of the EDAQ can be used as a measure of daily activity in practice and research in the Netherlands and German- speaking countries.

**Electronic supplementary material:**

The online version of this article (10.1007/s00296-020-04657-7) contains supplementary material, which is available to authorized users.

## Introduction

Patient-Reported Outcome Measures (PROMs) are used in clinical practice and research to identify patients with rheumatic and musculoskeletal conditions’ (RMDs) functional problems and evaluate the effectiveness of rehabilitation for these. Most commonly, daily activities in the International Classification of Functioning, Disability and Health (ICF) domains of communication, mobility, self-care and domestic life are assessed [1]. There is no single PROM of daily activities widely used by rheumatology health professionals (RHPs) in Europe. Those commonly used in rheumatoid arthritis (RA) research, for example, include between 10 and 21 activities [2–7]. In practice, RHPs often prefer non-validated daily activity checklists, including up to 55 activities [8], because they provide detailed information for individual treatment planning. However, such checklists lack reliability and validity to evaluate rehabilitation.

Consequently, a reliable, valid comprehensive daily activity PROM, including activities identified as problematic by people with RMDs, would be valuable: for treatment planning and evaluation; and in audit and research. The Evaluation of Daily Activity Questionnaire (EDAQ) was developed in Sweden, with women with RA, to meet these needs [9]. It takes patients 30 min to complete at home, allowing reflection on activity abilities. During rehabilitation, RHPs can quickly focus on identified problems, allowing more time for solutions.

The EDAQ has been extensively updated, culturally and linguistically validated and psychometrically tested in English. British men and women with RMDs identified new items and domains to include. It has been tested in eight RMDs: RA, ankylosing spondylitis (AS), osteoarthritis (OA), systemic lupus erythematosus, systemic sclerosis, chronic pain, chronic upper limb disorders and Primary Sjögren’s syndrome [8, 10–12]. Construct representation identified activities in the EDAQ rated as more difficult in RA require: greater overall physical demand, bilateral and fine hand use [13]. Content has been linked to the ICF [8] and ICF Core Sets for AS, OA, chronic widespread pain, low back pain and musculoskeletal conditions for post-acute care [11]. Over 80% of people with RMDs considered it the right length and helpful for discussing activity abilities with an RHP [10, 12]. The English EDAQ is also available online. Patients can complete it, store results to their user profile and e-mail them to their RHP [14]. Cross-cultural adaptation of the EDAQ into other languages would enable its use in other countries; and cross-country comparisons of patients’ rehabilitation needs and effects of rehabilitation on activities. To do this, cross-cultural invariance must be demonstrated, i.e. the EDAQ works in a consistent manner across language versions [15, 16].

The objectives of this study were to: linguistically validate and cross-culturally adapt the EDAQ for Dutch and German speakers with RA; test cross-cultural invariance across the Dutch, German and English versions of the EDAQ to ensure equivalent scaling; test the psychometric properties of the Dutch and German EDAQ; and further establish content validity in RA by linking the EDAQ to the ICF Core Set for RA.

## Methods

### Participants

Participants were recruited by health service staff identifying eligibility. Dutch-speakers were recruited from one hospital in the Netherlands. German-speakers were recruited from three hospitals in Switzerland and from Swiss, German and Austrian arthritis patient associations. Participants from patient associations volunteered following reading study information on associations’ websites and completing an eligibility screening form. As the Dutch and German language EDAQs were tested for cross-cultural adaptation and invariance with the English EDAQ, data from the earlier English study were included [10].

Participants were eligible if they: had a confirmed diagnosis of RA; were able to read, write and understand Dutch or German (as applicable); and had not (or were not about to) altered their disease-modifying medication regimen in the last 3 months (which could affect test–retest reliability).

Ethical approval was obtained, and all participants provided written informed consent.

### Phase 1: validation

#### Linguistic and cross-cultural validation

Recommended procedures were followed [17, 18]. Validation occurred in two stages. In stage one, two independent forward translations were made for each of the Dutch and German versions from the original Swedish EDAQ; these were synthesised by expert panels in each country, respectively; independent back translations were made from each language into Swedish; followed by synthesis by the Swedish language expert panel for each language to check for equivalence of meaning. In stage two: additional items developed for the English EDAQ were forward/backward translated as above into Dutch and German. Harmonization of the Dutch, German and English versions by the research teams then ensured equivalence.

Field testing of the Dutch and German EDAQs was conducted with people with RA in the Netherlands and Switzerland, respectively, using cognitive debriefing interviews [17, 19]. Participants completed the draft Dutch or German EDAQ at home and, within 2 weeks, were interviewed about comprehensibility, the relevance of the activities for people with RA and whether any important daily activities were missing. The results were discussed between the Dutch, German and English research teams. Further wording changes and additional items were agreed to ensure equivalence across these three versions.

#### Content validity

To further evaluate the content validity of the EDAQ, Part 2 items were systematically linked to the ICF Core Set for RA [20], using content linking rules [21].

### Phase 2: Psychometric testing

Participants were mailed a questionnaire booklet which collected data to describe the recruited population: age, gender, marital, educational and employment status, disease duration and RA disease-modifying medication, as well as the EDAQ and the measures described below. Two to three weeks later, participants were mailed the EDAQ to complete for a second time at home to evaluate test–retest reliability. Two reminders were sent for each mailing, as necessary.

#### Measurement instruments

The EDAQ includes three parts: Part 1 comprises 10 numerical rating scales (NRS) to assess symptom severity, mood and life satisfaction, each scored on a 0 (none) to 10 (severe) scale. Part 2 comprises 138 activities in 14 domains. Twelve can be combined into two Components: Self-Care (Eating and Drinking; In the Bathroom and Personal Care; Getting Dressed/Undressed; Cooking; Cleaning the House; Laundry and Clothes Care; Communication); and Mobility (Bathing and Showering; Moving Indoors; Moving and Transfers; Moving Outdoors and Shopping; Gardening and Household Maintenance). The other two domains are Caring; and Leisure, Hobbies and Social Activities. Items are scored on a 4-point scale assessing ability to perform daily activities (0 = no difficulty, 3 = unable to do). If the person would not normally perform that activity (for reasons other than health), there is a “not applicable” option. Each item is answered twice by rating performance without (Section A) and then with (Section B) ergonomic solutions (e.g., alternative methods, assistive devices, environmental modifications). In ICF terminology, section A relates to capacity and B to performance [1]. Items are summed to produce total scores for Sections A and B within each domain, with any score reductions between Sections A and B denoting the impact of ergonomic solutions on improving activity ability. If there are missing items within a domain, a total domain score cannot be calculated. Higher scores indicate greater activity limitations. The optional Part 3 includes a list of assistive devices and whether owned and used [10]. Part 3 was not tested as it is not used as an outcome measure.

The comparator health measures to assess concurrent validity were:

(i) The Health Assessment Questionnaire (HAQ): assessing ability to perform 20 daily activities rated on a 0-3 scale (0 = not at all difficult; 3 = unable to do) [22, 23]. These were summed to give a total score, as the HAQ20 does not score items worse if an assistive device is used [24]. Higher scores indicate greater activity limitations.
(ii) The Physical Function, Bodily Pain and Vitality (fatigue) scales of the Medical Outcomes Survey 36-item Short-Form version 2 (SF36v2), with norm-based scoring [6, 25]. Lower scores denote worse health states.
(iii) Hand pain: measured using a 11-point NRS of hand/wrist pain in the past week in during moderate activities (e.g., cooking a meal, doing housework/light gardening: 0 = no to 10 = severe).
(iv) RA Quality of Life scale (RAQoL): 30 items about QoL answered yes (= 1) or no (= 0), with yes items summed to give a total score. Higher scores indicate worse QoL [26].
(v) Perceived health status: using a 5-point NRS asking effects of their condition in the last month (1 = very good: no symptoms/no limitations in daily activities to 5 very poor: very severe symptoms/inability to carry out most activities).
(vi) Perceived change in health status: At Test 2 only, a 5-point NRS asking how much arthritis troubled them compared to when last completing the questionnaire (1 = much less to 5 = much more).

#### Sample size

As Rasch analysis was used to assess the invariance of the EDAQ Part 2 across language versions, a sample size of at least 150 for each language was necessary. This number was determined to ensure: a uniform distribution of patients across the construct of activity limitation; the precision of the estimate of both persons and items remains similar across the construct; and enough cases to test for invariance across groups. The sample does not need to be representative, as the mathematical model is independent of distribution, but it should have a good distribution across the activity domains [27]. At least, 79 sets of repeated responses were required to demonstrate that a test–retest correlation of 0.7 differed from a background correlation (constant) of 0.45, with 90% power at the 1% significance level. A test re-test correlation of 0.7 is deemed a minimum acceptable level [28].

### Statistical analyses

#### Cross-cultural adaptation and invariance

Rasch analysis is an iterative process of fitting data to the Rasch Measurement Model [29]. If the data meet the model expectations, then ordinal raw scores can be transformed into an interval level latent estimate [30]. Those expectations are associated with several assumptions underlying the model, namely stochastic ordering of items, local independence of items, unidimensionality and group invariance [31]. For unidimensionality, a *t* test of two estimates is made to ascertain if more than 5% of such estimates are different, or at least at the lower confidence interval for the proportion of different tests [32]. Rasch analysis of the English EDAQ Part 2 has already determined that total domain scores and component scores can be considered as unidimensional and total raw scores used [10]. Total domain and component scores can be converted to a Rasch metric when required for parametric analyses [33].

In cross-cultural adaptation, group invariance is crucial, as this determines if the adaptation has provided equivalent scaling, in this case across languages. Invariance is tested through Differential Item Functioning (DIF) [27]. As both the Dutch and German versions of the scale were made from the English version, invariance was tested from the English version for each, and across all three languages combined. The 12 domains of the two components of Self-Care and Mobility were used as testlets (as already determined in the earlier English Rasch analysis [10] and were the summed score of the items within each domain [34]. If local dependency remained across the domains these were further aggregated, as required. The analysis fits data to the Rasch model for each component (i.e. Self-Care and Mobility). at their respective domain levels. The RUMM2030 software was used [35].

#### Psychometric testing

The Statistical Package for the Social Sciences (SPSS) v25 was used for analyses [36], apart from linear weighted kappas, calculated using MedCalc [37]. As all measures consist of ordinal data, non-parametric statistical tests were used to assess the psychometrics, apart for intra-class correlation coefficients [ICC (2,1)] and sensitivity to change statistics, which were calculated using Rasch transformed data as interval data is required for these calculations [33]. Ordinal data are summarized as medians and inter-quartile ranges. Normality of Rasch transformed data was tested using the Kolmogorov–Smirnov test and data summarized using means and standard deviations.

*Test*–*retest reliability* EDAQ part 1 NRS were assessed using linear weighted kappas, as were individual Part 2 domain items. Agreement of ≥ 0.61 is good [38]. Part 2 domain total scores were assessed using: (a) Spearman’s correlations (with a correlation of ≥ 0.6 being strong [39]); and (b) ICC (2,1): two-way random consistency, average measures model, with ICC ≥ 0.75 considered excellent [40].

*Internal consistency* was assessed using Cronbach’s alpha, with results of ≥ 0.80 being good to excellent [39].

*Concurrent validity* of the Part 1 NRS and Part 2 domain total scores was assessed using Spearman’s correlations with measures of related constructs. For Part 1, this was the SF-36v2 sub-scales, except for Satisfaction with Life correlated with the RAQOL. For Part 2, this was the HAQ20, SF-36v2 sub-scales, RAQoL, Pain, Fatigue and Hand Pain NRS and Perceived Health Status.

*Discriminant validity* was assessed using Kruskal–Wallis tests to evaluate differences in scores between participants with different perceived health status groups.

*Sensitivity to change* was assessed by calculating Standard Error of Measurement (SEM) and the Minimal Detectable Change_95_ (MDC_95_). The formula used was: SEM = *s*√(1 − *r*), where *s* = the mean and standard deviation (SD) of Test 1 and Test 2 (retest), *r* = the reliability coefficient for the test, i.e. Pearson’s correlation co-efficient between Test and Test 2 values. Thereafter the MDC_95_ was calculated using the formula: MDC_95_ = SEM × √2 × 1.96 [41, 42].

*Floor and ceiling effects* were considered present if > 15% of participants achieved either the lowest or highest scores in the 14 EDAQ Part 2 domains [43].

## Results

### Phase 1: validation

The only difficulties encountered during translation were identifying names for some assistive devices in part 3. This was overcome with photographs and local therapists providing correct names. Cognitive debriefing interviews were undertaken with six Dutch- and five German-speaking participants. Average time to complete all three parts of the EDAQ was 30 (SD 8) minutes. Activities in the EDAQ Part 2 were considered culturally relevant by both Dutch and German participants. Some additional activities were added to existing items: use of smartphones; laptop/tablet (e.g. iPad) to the Communication domain; bicycling to the Leisure, Hobbies and Social Activities domain. Ten considered it easy/partially easy to complete, with five highlighting the importance of carefully reading instructions. Eight commented that Part 2 (activities) was most relevant and three that Part 3 (assistive devices) least relevant because those participants had few or no assistive devices. Seven considered the EDAQ included the right range and number of activities. However, four thought there were too many. All 11 participants considered the EDAQ would capture the difficulties they face daily and enable discussions with rehabilitation health professionals.

#### Linking to the ICF Core Set for RA

The Part 2 EDAQ had good content validity, with 28/33 activities from the Communication, Mobility, Self-Care and Domestic Life items of the RA Core Set included. The five Core Set activities not included were not specific daily activities: carrying out daily routine (d230); Interpersonal family (d760) and intimate (d770) relationships; and Major Life areas: remunerative (d850) and other work/employment (d859). (see Supplementary Table S1).

### Phase 2: psychometric testing

#### Participants

The sample consisted of 252 Dutch-speaking people from the Netherlands and 163 German-speaking people (87 from Switzerland, 70 from Germany and 6 from Austria). Their language group-specific demographic characteristics and disease duration are shown in Table 1 and health status in Table 2. Demographic and health data for the English-speaking participants (*n* = 383), included in the Rasch analysis, is also shown. The Dutch sample, compared to the German and English-speaking samples, were older with: more men; shorter disease duration; less educational experience; fewer on biologics; and better health status.

**Table 1:** Rheumatoid Arthritis participants’ demographic characteristics completing the Evaluation of Daily Activity Questionnaire Dutch, German and English versions

	RA Dutch (*n* = 252)	RA German (*n* = 163)	RA English (*n* = 369)
Age: years: mean (SD)	65.16 (13.45)	52.84 (14.94)	60.38 (11.19)
Gender M:F; *n* (%)	93:155 (38:62%)	18:145 (11:89%)	97:286 (25:75%)
Condition duration in years: mean (SD)	11.75 (9.93)	15.73 (12.12).	13.20 (10.70)
Marital status: *n* (%)			
- Married/living with a partner	179 (71%)	102 (62.58%)	276 (72%)
Living status: *n* (%)			
- With family/significant other	177 (70%)	107 (66%)	292 (76%)
- Children living at home *n* (%):	20 (8%)	21 (13%)	92 (24%)
Employment status: *n* (%)			
- Paid employment	68 (27%)	85 (52%)	118 (31%)
Education level: *n* (%)			
- Secondary education only	189 (75%)	94 (58%)	174 (45%)
Current medication: n (%)			
- DMARDs	163 (65%)	56 (34%)	193 (50%)
- Biologic drugs	43 (17%)	80 (49%)	161 (42%)
Perceived Health: n (%)	(*n* = 239)	(*n* = 162)	(*n* = 378)
- Very good/good (1-2)	144 (60%)	82 (51%)	135 (35%)
- Fair (3)	79 (33%)	60 (37%)	146 (38%)
- Poor/Very Poor (4-5)	16 (7%)	20 (12%)	97 (25%)
Numbers answering Test 2	155	107	–

*DMARDS* disease modifying anti-rheumatic drugs

**Table 2:** Health status measures in the Evaluation of Daily Activity Questionnaire Dutch, German and English versions in Rheumatoid Arthritis: median (inter-quartile range)

Measure (score range)	RA Dutch (*n* = 252)	RA German (*n* = 163)	RA English (*n* = 369)
HAQ20 (0–60)	4 (1–15)	7 (2–14)	13 (4–24)
SF36v2 physical function (0–100)	44.51 (32.66–51.80)	44.15 (36.01–51.80)	34.57 (25.01–44.15)
SF36v2 bodily pain (0–100)	46.68 (38.61–55.55)	42.24 (38.21–50.71)	38.21 (34.18–46.68)
SF36v2 vitality (0–100)	52.60 (46.66–58.54)	49.63 (40.72–52.60)	40.72 (31.80–49.63)
SF36v2 mental health (0–100)	53.48 (43.02–58.72)	50.87 (43.02–56.10)	50.87 (40.40–56.10)
RAQoL (0–30)	5.50 (2–12)	8 (4–15)	13 (5–20)
Hand pain (0–100)	3 (1–5)	3 (1–5)	5 (2–7)

*HAQ* Health Assessment Questionnaire, *SF36v2* Short Form 36v2, *RAQoL* Rheumatoid Arthritis Quality of Life scale, *Higher scores* better in SF36v2, *Lower scores* better in HAQ20 and RAQOL

#### Rasch analysis

Fit of the data to the Rasch model for each component and language is shown in Table 3. In most cases, adequate fit to the model was observed, along with unidimensionality (ideal values are shown at the bottom of the table). Overall, there was only sporadic DIF at the domain level, with no consistent pattern. Where DIF did occur, the magnitude of difference in the mean locations of groups defined by their level of disability was small. It was always present in only one of the groups, always at the margins, that is either, ‘no/little’ self- care disability or ‘highest’ self- care disability. When the domains were further grouped into larger super items, the DIF was no longer present, suggesting that at the test level, DIF would cancel out. There was little DIF observed by language indicating that the hierarchical ordering of domains remained the same across the language pairwise analyses. Only for the combined analysis of the domains within the mobility component was it necessary to merge the domains into super items to obtain fit (a conditional test of fit is provided at this level). Rasch transformation tables converting component raw scores to interval scales are shown in Supplementary Tables S3 and S4.

**Table 3:** Fit of Evaluation of Daily Activity Questionnaire components to the Rasch model: Dutch, German and English versions

	Chi-Square	DF	P	Reliability	Unidimensionality % *t* test significant	Lower CI of *t* test	*N*
*Self-care*							
Dutch	37.2	28	0.11	0.81	6.8	4.0	220
German	11.8	14	0.62	0.89	6.7	3.2	150
English	35.9	42	0.74	0.91	4.6	2.2	369
Dutch and English	70.6	63	0.24	0.88	4.7	2.9	589
German & English	59.4	63	0.61	0.90	3.9	2.0	519
Combined	67.8	63	0.32	0.88	4.8	3.1	739
*Mobility*							
Dutch	44.6	25	0.01	0.80	3.2	0.3	220
German	16.0	10	0.10	0.88	6.5	3.0	150
English	16.6	25	0.89	0.88	4.0	1.6	369
Dutch and English	18.7	18	0.41	0.86	2.3	− 0.6	589
German and English	40.8	35	0.23	0.88	5.5	3.5	519
Combined	78.8	83	0.60 ~	0.87	3.3	1.1	739
*Ideal values*			> 0.05*	> 0.85	<5 .0	< 5.0	

*DF* degrees of freedom, *CI* = confidence interval, ~ conditional test of fit

*Bonferroni adjusted = 0.008 within each component

#### Test–retest reliability

Part 1: the 10 NRS had moderate to good reliability (Table 4).

**Table 4:** Evaluation of Daily Activity Questionnaire Part 1: test–retest reliability and concurrent validity in the Dutch (*n* = 252) and German (*n* = 163) versions

Part 1 EDAQ numeric rating scale: (0–10)	Test 1 Median (IQR)	Test 2 Median (IQR)	Test–retest reliability linear weighted kappa	Comparator measures (*r*_s_)
*1: Disease activity*				
Dutch	3.5 (2–6)	4 (2–6)	0.42	− 0.45^a^***
German	4 (2–6)	4 (2–6)	0.43	− 0.43^a^***
*2: Mood*				
Dutch	1 (0–4)	2 (0–4)	0.40	− 0.64^d^***
German	3 (2–6)	3 (2–6)	0.46	− 0.57^d^***
*3: Pain when resting*				
Dutch	2 (1–4)	2 (1–4)	0.50	− 0.68^b^***
German	2 (1–5)	3 (1–5)	0.48	− 0.63^b^***
*4: Pain when moving*				
Dutch	3 (1–6)	3 (1.75–5)	0.60	− 0.73^b^***
German	4 (2–6)	4 (2–6)	0.52	− 0.73^b^***
*5: Stiffness*				
Dutch	3 (1–6)	3 (2–5)	0.58	− 0.55^a^***
German	3 (1–5)	3 (1–6)	0.60	− 0.52^a^***
*6: Joint movement limitations*				
Dutch	3 (1–6)	3 (1–6)	0.64	− 0.64^a^***
German	4 (2–6)	3 (2–6)	0.60	− 0.69^a^***
*7: Fatigue*				
Dutch	4 (2–7)	4 (1.75–6)	0.60	− 0.72^c^***
German	5 (3–7)	5 (2–7)	0.59	− 0.72^c^***
*8: Worry*				
Dutch	3 (1–5)	2 (1–4)	0.59	− 0.52^d^***
German	2 (1–5)	2 (1–5)	0.49	− 0.67^d^***
*9: Sleep*				
Dutch	2 (0–5)	2 (1–5)	0.62	− 0.48^b^***
German	3 (1–6)	3 (1–6)	0.69	− 0.42^b^***
*10: Satisfaction with life*				
Dutch	1 (0–3)	1 (0–3)	0.53	0.55^e^***
German	3 (1–5)	3 (1–6)	0.51	0.57^e^***

For all EDAQ Part 1 scales and RAQOL, higher scores are worse; SF36v2 higher scores are better

*r*_*s*_ Spearman’s correlation coefficient

***p = < 0.001; **p < 0.01; *p < 0.05

^a^Short Form F36v2 Physical Function scale

^b^Short Form 36v2 Pain scale

^c^Short Form 36 v2 Vitality scale

^d^SF36v2 Mental Health scale

^e^RAQOL. Not all scales had directly applicable comparator measures

Part 2: correlations between test 1 and 2 domain scores were good or very good (*r*_s_ = 0.75 to 0.93), apart from the Caring domain which had only moderate correlations (Table 5). The domains’ intra-class correlations were excellent [ICC (2,1) = 0.90 to 0.97], apart from Gardening and Household Maintenance (Dutch) which was lower at 0.77 (Table 5). Linear weighted kappa scores for individual items in each domain were mainly moderate to good (0.20–0.75 Dutch; 0.41–0.82 German version) (Supplementary Table 2).

**Table 5:** Evaluation of Daily Activity Questionnaire Part 2: Section A and B median (inter-quartile range) scores, internal consistency, test retest reliability and sensitivity to change Dutch (*n* = 252) and German (*n* = 163) versions

EDAQ domain (score range)	Test 1 Section A score	Test 1 A (α)	Test 1 Section B score	Test 2 Section A score	Test 2 Section B score	Test- retest Section A r_s_	Test–retest Section B r_s_	ICC(2,1) (95% CI)	SEM	MDC_95_
*1: Eating and drinking (0–33)*										
Dutch	4 (1–8)	0.91	4 (1–7.25)	5 (1–9)	4 (1–7)	0.89***	0.87***	0.94 (0.92, 0.96)	0.66	1.83
German	6 (3–10.5)	0.91	5 (2–9)	6 (2–10)	4 (1–7)	0.81***	0.83***	0.91 (0.87, 0.94)	1.17	3.24
*2: In the bathroom and personal care (0–36)*										
Dutch	1 (0–3)	0.85	1 (0–3.75)	1 (0–3)	1 (0–3)	0.81***	0.81***	0.90 (0.86, 0.93)	0.82	2.26
German	2 (0–6)	0.90	2 (0–5)	2 (0–6)	2 (0–6)	0.83***	0.81***	0.91 (0.87, 0.94)	0.97	2.68
*3: Dressing (0–33)*										
Dutch	2 (0–7)	0.94	2 (0–7)	2 (0–7.5)	2 (0–7.25)	0.78***	0.77***	0.90 (0.86, 0.93)	1.27	3.53
German	4 (1–9)	0.95	3 (1–9)	4 (1–9)	3 (1–9)	0.88***	0.86***	0.94 (0.91, 0.96)	1.17	3.25
*4: Bathing and showering (0–33)*										
Dutch	2 (0–7)	0.93	2 (0–6)	2 (0–7.75)	2 (0–7)	0.83***	0.79***	0.92 (0.89, 0.95)	1.23	3.42
German	4 (1–9)	0.92	3 (1–8)	4 (0–10)	4 (0–9)	0.88***	0.85***	0.94 (0.91, 0.96)	1.20	3.31
*5: Cooking (0–42)*										
Dutch	2 (0–9)	0.94	2 (0–8)	2 (0–6.25)	2 (0–6)	0.82***	0.80***	0.92 (0.89, 0.95)	1.33	3.68
German	7 (1.5–13)	0.96	7 (1–12)	7 (1–14)	7 (1–13)	0.91***	0.89***	0.96 (0.94, 0.97)	0.83	2.30
*6: Moving indoors (0–36)*										
Dutch	5 (1–11)	0.92	5 (1–11)	4 (1–10)	4 (1–9)	0.86***	0.86***	0.93 (0.90, 0.95)	1.14	3.16
German	7 (3–11)	0.89	7 (3–11)	7 (3–12)	7 (3–11.75)	0.90***	0.89***	0.95 (0.93,0.97)	0.68	1.89
*7: Cleaning the house (0–27)*										
Dutch	4 (1–10)	0.95	4 (1–10)	4 (1–8)	4 (1–8)	0.84***	0.84***	0.93 (0.90, 0.95)	1.00	2.78
German	7 (2–13)	0.95	7 (2–13)	7 (2–12)	7 (2–12)	0.93***	0.92***	0.97 (0.95,0.98)	0.57	1.58
*8: Laundry and clothes care (0–27)*										
Dutch	1 (0–6)	0.92	1 (0–6)	1 (0–5)	1 (0–5)	0.80***	0.80***	0.90 (0.86, 0.93)	1.17	3.23
German	4 (1–9)	0.94	3 (1–9)	4 (0–10)	3 (0–10)	0.88***	0.88***	0.94 (0.91,0.96)	0.89	2.46
*9: Moving and transfers (0–18)*										
Dutch	2 (0–5)	0.94	2 (0–5)	2 (0–5)	2 (0–5)	0.85***	0.85***	0.92 (0.89, 0.94)	0.72	2.00
German	3 (1–6)	0.85	3 (1–6)	3 (0–6)	3 (0–6)	0.82***	0.81***	0.90 (0.85, 0.92)	0.92	2.55
*10: Communication (0–18)*										
Dutch	1 (0–3)	0.79	1 (0–3)	0 (0–3)	0 (0–3)	0.75***	0.75***	0.90 (0.85, 0.92)	0.45	1.25
German	2 (0–4)	0.87	1 (0–4)	1 (0–4)	1 (0–3)	0.83***	0.83***	0.91 (0.87,0.94)	0.61	1.71
*11: Moving outdoors and shopping (0–39)*										
Dutch	6 (2–11)	0.92	5 (2–10.77)	5 (1–11)	5 (1–11)	0.92***	0.92***	0.95 (0.93, 0.97)	0.95	2.63
German	7 (2–13)	0.92	7 (2–11)	7 (2–13)	7 (1–11)	0.91***	0.90***	0.95 (0.92, 0.96)	0.92	2.55
*12: Gardening and household maintenance (0–21)*										
Dutch	2 (0–6)	0.89	2 (0–6)	1 (0–3)	1 (0–3)	0.66***	0.66***	0.78 (0.68, 0.84)	2.80	7.77
German	5 (1–10)	0.93	5 (1–10)	5 (1–10)	5 (1–10)	0.93***	0.90***	0.95 (0.92,0.96)	0.93	2.58
*13: Caring (0–27)*										
Dutch	0 (0–1)	0.89	0 (0–1)	0 (0–0)	0 (0–0)	0.56***	0.56***	–	–	–
German	0 (0–5)	0.96	0 (0–5)	0 (0–3)	0 (0–3)	0.80***	0.80***	–	–	–
*14: Leisure, hobbies and social activities (0–27)*										
Dutch	2 (0–4)	0.86	1.5 (0–4)	1 (0–3)	1 (0–3)	0.75***	0.75***	–	–	–
German	3 (1–8)	0.89	3 (1–8)	3 (1–7)	3 (1–7)	0.83***	0.83***	–	–	–

*α* Cronbach’s alpha, *r*_*s*_ Spearman’s correlation coefficient, *ICC* intra-class correlation coefficient, *SEM* Standard Error of Measurement, *MDC*_*95*_ Minimum Detectable Change_95_

***p ≤ 0.001; **p < 0.01; *p < 0.05

#### Internal consistency

Cronbach’s alpha values for the 14 domains were all excellent (≥ 0.85) apart from Communication (0.79) in the Dutch version (Table 5). All domains in both languages, therefore, had values consistent with group use (i.e. ≥ 0.7), and most with individual use (i.e. ≥ 0.85) [28]. Each domain can be used as a stand-alone measure, as well as collectively within the two components of Self-Care and Mobility.

#### Concurrent validity

In EDAQ Part 1, there were moderate to strong correlations between NRS and SF36v2 Mental Health, Vitality and Bodily Pain scales and RAQOL, as relevant (*r*_s_ =  − 0.42 to − 0.73) Table 4).

In EDAQ Part 2, most domains correlated strongly with physical function measures: HAQ20 (*r*_s_ = 0.65 to 0.87), apart from Gardening and Household Maintenance (Dutch) and Caring (Dutch and German) which were moderate; and SF36v2 Physical Function (*r*_s_ = − 0.61 to − 0.87), apart from Bathroom and Personal Care, Communication, Gardening and Household Maintenance which were moderate and Caring which was weak in the Dutch version and moderate in the German version.

The Part 2 domains correlated moderately/strongly with pain symptoms: SF36v2 Bodily Pain (*r*_s_ = − 0.44 to − 0.67); Hand Pain (*r*_s_ = 0.43–0.64); and Pain (*r*_s_ = 0.46–0.66) apart from Gardening and Household Maintenance (Dutch) which was weak. Generally, EDAQ domains correlated only moderately with fatigue symptoms in the Dutch version (*r*_s_ = − 0.35 to − 0.75) and weakly to moderately in the German version (*r*_s_ = 0.26–0.42). The RAQoL correlated moderately to strongly with EDAQ domains (*r*_s_ = 0.50–0.83). Perceived Health State was moderately to strongly correlated (*r*_s_ = 0.47–0.65). Apart from being moderately correlated with physical function measures, the Caring domain was weakly correlated with other measures (Table 6).

**Table 6:** Evaluation of Daily Activity Questionnaire Part 2: concurrent validity with comparator measures (Dutch *n* = 252; German *n* = 163)

EDAQ domain section A (score range)	HAQ20 (*r*_s_)	SF36v2: PF (*r*_s_)	SF36v2: bodily pain (*r*_s_)	SF36v2: vitality (*r*_s_)	Quality of life: RAQOL (*r*_s_)	Pain NRS (*r*_s_)	Fatigue NRS (*r*_s_)	Hand pain NRS (*r*_s_)	Perceived health (*r*_s_)
*1: Eating and drinking (0–33)*									
Dutch	0.77***	− 0.63***	− 0.58***	− 0.50***	0.69**	0.59***	0.60***	0.61***	0.49***
German	0.77***	− 0.70***	− 0.58***	− 0.30***	0.55***	0.48***	0.26***	0.50***	0.52***
*2: In the bathroom and personal care (0–36)*									
Dutch	0.67***	− 0.57***	− 0.47***	− 0.40***	0.65***	0.52***	0.56***	0.50***	0.55***
German	0.81***	− 0.70***	− 0.53***	− 0.34***	0.57***	0.52***	0.35***	0.44***	0.50***
*3: Dressing (0–33)*									
Dutch	0.75***	− 0.64***	− 0.59***	− 0.48***	0.65***	0.56***	0.57***	0.58***	0.53***
German	0.79***	− 0.73***	− 0.56***	− 0.27***	0.55***	0.57***	0.28***	0.43***	0.53***
*4: Bathing and showering (0–33)*									
Dutch	0.86***	− 0.79***	− 0.61***	− 0.54***	0.76***	0.61***	0.65***	0.62***	0.55***
German	0.83***	− 0.79***	− 0.53***	− 0.33***	0.58***	0.56***	0.38***	0.46***	0.55***
*5: Cooking (0–42)*									
Dutch	0.82***	− 0.73***	− 0.61***	− 0.55***	0.74***	0.58***	0.63***	0.61***	0.54***
German	0.87***	− 0.81***	− 0.60***	− 0.40***	0.68***	0.58***	0.38***	0.52***	0.59***
*6: Moving indoors (0–36)*									
Dutch	0.85***	− 0.83***	− 0.66***	− 0.60***	0.83***	0.65***	0.75***	0.64***	0.54***
German	0.83***	− 0.84***	− 0.63***	− 0.36***	0.65***	0.60***	0.37***	0.51***	0.64***
*7: Cleaning the house (0–27)*									
Dutch	0.79***	− 0.76***	− 0.61***	− 0.52***	0.74***	0.52***	0.59***	0.57***	0.52***
German	0.87***	− 0.83***	− 0.59***	− 0.30***	0.63***	0.54***	0.32***	0.52***	0.56***
*8: Laundry and clothes care (0–27)*									
Dutch	0.75***	− 0.68***	− 0.56***	− 0.51***	0.65***	0.46***	0.55***	0.52***	0.47***
German	0.86***	− 0.76***	− 0.53***	− 0.31***	0.57***	0.48***	0.32***	0.49***	0.52***
*9: Moving and transfers (0–18)*									
Dutch	0.78***	− 0.66***	− 0.60***	− 0.62***	0.77***	0.61***	0.68***	0.60***	0.56***
German	0.77***	− 0.76***	− 0.60***	− 0.42***	0.68***	0.64***	0.42***	0.50***	0.64***
*10: Communication (0–18)*									
Dutch	0.65***	− 0.55***	− 0.52***	− 0.45***	0.63***	0.54***	0.51***	0.59***	0.48***
German	0.73***	− 0.61***	− 0.49***	− 0.38***	0.55***	0.46***	0.33***	0.44***	0.52***
*11: Moving outdoors and shopping (0–39)*									
Dutch	0.88***	− 0.87***	− 0.67***	− 0.59***	0.83***	0.63***	0.67***	0.60***	0.59***
German	0.87***	− 0.87***	− 0.65***	− 0.41***	0.71***	0.66***	0.42***	0.52***	0.65***
*12: Gardening and household maintenance (0–21)*									
Dutch	0.51***	− 0.54***	− 0.44***	− 0.35***	0.50***	0.39***	0.45***	0.46***	0.31***
German	0.76***	− 0.79***	− 0.57***	− 0.33***	0.63***	0.53***	0.33***	0.47***	0.57***
*13: Caring (0–27)*									
Dutch	0.31**	− 0.22**	− 0.22**	− 0.16**	0.28***	0.18**	0.17**	0.24***	0.16*
German	0.42***	− 0.40***	− 0.31***	− 0.10	0.25**	0.37***	0.14	0.26**	0.27***
*14: Leisure, hobbies and social activities (0–27)*									
Dutch	0.76***	− 0.64***	− 0.65***	− 0.55***	0.71***	0.57***	0.60***	0.60***	0.57***
German	0.76***	− 0.77***	− 0.58***	− 0.40***	0.68***	0.55***	0.41***	0.47***	0.60***

***p = < 0.001; **p < 0.01; *p < 0.05

#### Discriminant validity

There were significant differences in most Part 2 domain scores (*p* < 0.01), except for the Dutch Caring domain (*p* = 0.27), as many participants reported not performing Caring activities (Supplementary Table 7).

#### Sensitivity to change

Part 2 MDC_95_ domain scores ranged from 1.25 to 3.68 apart from Gardening and Household Maintenance which was higher (7.77) for the Dutch EDAQ (Table 5).

#### Floor and ceiling effects

In the Dutch EDAQ all Part 2 domains, except Move Outdoors, had floor effects. For the German EDAQ all domains, except Eating, Move Indoors, Cleaning, Move Outdoors had floor effects. There were no celling effects in either version.

## Discussion

This study has produced linguistically validated and culturally relevant Dutch and German versions of the EDAQ which are now available for use. No changes were needed to be culturally relevant, with only a few additions necessary to further update content across language versions. In field testing, both versions were acceptable to participants, despite their length, and considered as helpful in improving communication about their activity limitations with health professionals. Both versions demonstrated good psychometric properties in people with RA from the Netherlands and Switzerland, Germany and Austria, respectively. They meet most of the recommendations for PROMs of the United States Food and Drug Administration [44] and the Consensus-based Standards for the selection of health Measurement Instruments (COSMIN) checklist [45, 46], although responsiveness still needs evaluating in a clinical trial or setting. Consequently, the EDAQ can also be used in research and audit, as well as clinical practice.

In terms of validity, the EDAQ Part 2 has good content validity covering most of the items in the Activities and Participation component of the ICF Core Set for RA. Good construct validity was demonstrated by satisfying Rasch model expectations. As at domain level no DIF was observed, raw scores for items in each component can be summed to create both total domain and component scores. Data can also be pooled across countries/languages and comparative studies of EDAQ data conducted.

Concurrent validity of the Dutch and German versions of the EDAQ reflected previous studies in the English EDAQ with people with RA and other RMDs, with strong correlations with physical function, moderate to strong with pain and moderate with fatigue [10, 12]. In all three language versions, the Caring domain had only weak correlations with other measures, predominantly because few had caring responsibilities. Further research is needed to test this domain in samples with a larger number of parents with young children to test validity for those with caring responsibilities. Internal consistency and test–retest reliability were similar to the English EDAQ results, and indicate the EDAQ can be used for both group and individual measurement in RA. As each domain is valid and reliable, health professionals can choose which domains to use in clinical practice. For example, the client may complete all the EDAQ initially. If their rehabilitation focuses only on selected domains, then at follow-up the client need only complete those domains.

A strength of this study is that it included a large sample of people with RA, recruited from both out-patient clinics and patient associations. Recruitment was from three German-speaking countries. Two German versions have been developed: one for Switzerland, and one for Germany and Austria, as there are minor differences in written German. Although the English version has been tested in eight RMDs, the Dutch and German EDAQs have only been tested in RA. Further research is needed to establish whether the Dutch and German EDAQs are reliable and valid in other RMDs. This would allow the EDAQ to be used across these language versions/countries in a wide variety of RMDs commonly treated in rheumatology departments and in other settings.

The limitations of this study are that, in Phase 2, the acceptability and utility of the EDAQ were not investigated, as in the English studies, although Phase 1 participants endorsed these. Additionally, floor effects were observed across most domains (particularly the Dutch sample). This is likely because the Dutch sample included a higher proportion of men, compared to the German and English samples. Men with RA tend to have fewer daily activity difficulties than women, predominantly because of their stronger grip force [47]. Additionally, in this Dutch sample more reported being in good health.

In conclusion, the Dutch and German EDAQs are valid, reliable measures of activity limitations which can be used with people with RA. Either the whole EDAQ, the Self-Care/Domestic Life or Mobility components or the individual domains can be used in clinical practice to identify client’s daily activity difficulties, facilitate discussion to find solutions, and evaluate the outcome. Equivalence between these and the English language version means that data from these different versions can be combined.

## Electronic supplementary material

Below is the link to the electronic supplementary material.

Supplementary material 1 (DOCX 69 kb)

## Data Availability

The Dutch and German EDAQ de-identified Test 1 datasets are available on the Figshare data repository: doi.0.6084/m9.figshare.12609689. Further data requests should be submitted to the corresponding author for consideration. Access to de-identified datasets may be granted following review. The EDAQ, EDAQ-D, EDAQ-NL and EDAQ Manualv3 are available for free download from the University of Salford Institutional Repository: http://usir.salford.ac.uk/id/eprint/30752; and/46269/46275/and/46276).
