# Supplementary material for: Cross-cultural adaptation and psychometric testing of the Dutch and German versions of the Evaluation of Daily Activity Questionnaire in people with rheumatoid arthritis
Source: Rheumatol Int. 2020 Jul 26;41(5):951–64. doi: 10.1007/s00296-020-04657-7 (PMC8019417; doi:10.1007/s00296-020-04657-7)
Supplement: Supplementary file 1 — Supplementary material 1 (DOCX 69 kb) [file 296_2020_4657_MOESM1_ESM.docx]

**Supplementary Materials**

Alison Hammond ^1,^ Jorit Meesters ^2^, Karin Niedermann ^3,^ Alan Tennant ^4^, Thea Vliet Vlieland ^5^, Sarah Tyson ^6^, Ulla Nordenskiold ^7^

Cross-cultural adaptation and psychometric testing of the Dutch and German versions of the Evaluation of Daily Activity Questionnaire in people with rheumatoid arthritis.

Rheumatology International (2020)

**Supplementary Table S1:** **Evaluation of Daily Activity Questionnaire Part 2 content linked to the International Classification of Function Core Set for Rheumatoid Arthritis.**

| **ICF Code:** | **ICF Category Title** | **Measured by Items within EDAQ Domains:** |
| --- | --- | --- |
| d170 | Writing | 10 Communication |
| d230 | Carrying out daily routine |  |
| d360 | Using communication devices and techniques | 10 Communication |
| d410 | Changing basic body position | 2 Bathroom and Personal Care  4 Bathing and Showering  5 Cooking  6 Moving Indoors  9 Moving and Transfers  11 Moving Outdoors and Shopping |
| d415 | Maintaining a body position | 6 Moving Indoors  9 Moving and Transfers |
| d430 | Lifting and carrying objects | 5 Cooking  6 Moving Indoors  11 Moving Outdoors and Shopping |
| d440 | Fine hand use | 1 Eating and Drinking  2 Bathroom and Personal Care  3 Dressing  4 Bathing and Showering  5 Cooking  6 Moving Indoors  7 Cleaning the House  8 Laundry and Clothes Care  9 Moving and Transfers  10 Communication  11 Moving Outdoors and Shopping  12 Gardening and Household Maintenance  14 Leisure and Social Activities |
| d445 | Hand and arm use | 1 Eating and Drinking  2 Bathroom and Personal Care  3 Dressing  4 Bathing and Showering  5 Cooking  6 Moving Indoors  7 Cleaning the House  8 Laundry and Clothes Care  11 Moving Outdoors and Shopping  12 Gardening and Household Maintenance |
| d449 | Carrying, moving and handling objects, other specified and unspecified | 5 Cooking  6 Moving Indoors  11 Moving Outdoors and Shopping |
| d450 | Walking | 6 Moving Indoors  11 Moving Outdoors and Shopping |
| d455 | Moving around | 6 Moving Indoors11 Moving Outdoors and Shopping |
| d460 | Moving around in different locations | 6 Moving Indoors  11 Moving Outdoors and Shopping |
| d465 | Moving around using equipment | 11 Moving Outdoors and Shopping |
| d470 | Using transportation | 11 Moving Outdoors and Shopping |
| d475 | Driving | 11 Moving Outdoors and Shopping |
| d510 | Washing oneself | 2 Bathroom and Personal Care  4 Bathing and Showering |
| d520 | Caring for body parts | 2 Bathroom and Personal Care  4 Bathing and Showering |
| d530 | Toileting | 2 Bathroom and Personal Care |
| d540 | Dressing | 3 Getting Dressed and Undressed |
| d550 | Eating | 1 Eating and Drinking |
| d560 | Drinking | 1 Eating and Drinking |
| d570 | Looking after one`s health | Partly in 14 Leisure (item 5 Physical activities) |
| d620 | Acquisition of goods and services | 11 Moving Outdoors and Shopping |
| d630 | Preparing meals | 5 Cooking |
| d640 | Doing housework | 7 Cleaning the House  8 Laundry and Clothes Care |
| d660 | Assisting others | 13 Caring |
| d760 | Family relationships |  |
| d770 | Intimate relationships |  |
| d850 | Remunerative employment |  |
| d859 | Work and employment, other specified and unspecified |  |
| d910 | Community life | 14 Leisure and Social Activities |
| d920 | Recreation and leisure | 14 Leisure and Social Activities |

**Supplementary Table S2: Evaluation of Daily Activity Questionnaire Part 2 domain item test-retest reliability: linear weighted kappa coefficients.**

| **Domain 1: Eating and Drinking** | Dutch  n = 252 | German  n=163 |
| --- | --- | --- |
| 1. Lift a glass | 0.75 | 0.47 |
| 1. Lift a cup | 0.68 | 0.46 |
| 1. Use a knife and fork | 0.60 | 0.65 |
| 1. Slice food (e.g. bread, cheese) | 0.58 | 0.64 |
| 1. Get the milk out of the fridge | 0.44 | 0.67 |
| 1. Open a milk carton/ plastic bottle and pour out | 0.54 | 0.64 |
| 1. Open a bottle top (e.g. lager) | 0.46 | 0.57 |
| 1. Open a screw top jar or bottle | 0.61 | 0.61 |
| 1. Open a tin or ring pull can | 0.63 | 0.62 |
| 1. Open a packet/pouch | 0.49 | 0.62 |

| **Domain 2: In the Bathroom and Personal Care** | Dutch  n = 252 | German  n=163 |
| --- | --- | --- |
| 1. Get on and off the toilet | 0.60 | 0.62 |
| 1. 2. Wipe yourself with toilet paper/ clean self below | 0.61 | 0.62 |
| 1. Use tampons /suppositories | 0.36 | 0.57 |
| 1. Flush the toilet | 0.38 | 0.52 |
| 1. Arrange your clothes after going to toilet | 0.52 | 0.66 |
| 1. Wash your hands | 0.28 | 0.45 |
| 1. Brush and comb your hair | 0.59 | 0.70 |
| 1. Brush your teeth | 0.70 | 0.63 |
| 1. Use a tube of toothpaste | 0.40 | 0.60 |
| 1. Open a medicine bottle/ blister pack | 0.57 | 0.63 |
| 1. Do your make-up or shave | 0.65 | 0.47 |
| 1. Put on jewellery/watch | 0.47 | 0.70 |

| **Domain 3: Dressing** | Dutch  n = 252 | German  n=163 |
| --- | --- | --- |
| 1. Put on / take off a coat | 0.24 | 0.68 |
| 1. Pull clothes over your head | 0.59 | 0.63 |
| 1. Put on front-opening clothes | 0.63 | 0.62 |
| 1. Do up/undo buttons | 0.64 | 0.69 |
| 1. Pull clothes over your feet | 0.51 | 0.68 |
| 1. Do up /undo zips | 0.51 | 0.58 |
| 1. Put on tights/socks | 0.64 | 0.68 |
| 1. Take shoes/ boots on and off | 0.55 | 0.61 |
| 1. Tie shoelaces | 0.59 | 0.77 |
| 1. Put on/take off gloves | 0.49 | 0.62 |
| 1. Fasten clothes at the back | 0.58 | 0.67 |

| **Domain 4. Bathing and Showering** | Dutch  n = 252 | German  n=163 |
| --- | --- | --- |
| 1. Get in and out of the bath | 0.49 | 0.65 |
| 1. Shower whilst standing | 0.67 | 0.60 |
| 1. Use shower controls /bath temperature mixers | 0.46 | 0.47 |
| 1. Turn taps *(any in the home)* | 0.48 | 0.61 |
| 1. Wash your back and neck | 0.65 | 0.71 |
| 1. Dry your back and neck | 0.63 | 0.66 |
| 1. Wash and dry your feet | 0.60 | 0.64 |
| 1. Wash your hair | 0.73 | 0.72 |
| 1. Style/ blow-dry your hair | 0.58 | 0.71 |
| 1. Cut/file your fingernails | 0.61 | 0.70 |
| 1. Take care of feet | 0.60 | 0.65 |

| **Domain 5. Cooking** | Dutch  n = 252 | German  n=163 |
| --- | --- | --- |
| 1. Stand while working in the kitchen | 0.60 | 0.74 |
| 1. Set table/ carry plates, cups etc. | 0.54 | 0.60 |
| 1. Peel and chop vegetables | 0.66 | 0.73 |
| 1. Carry a full pan to/ from cooker | 0.67 | 0.75 |
| 1. Drain water from a saucepan (e.g. vegetables, pasta) | 0.58 | 0.74 |
| 1. Remove heavy items (e.g. bag of sugar) from top cupboards | 0.54 | 0.62 |
| 1. Baking (e.g. cakes, bread, pastry) | 0.51 | 0.61 |
| 1. Take things in/out of oven | 0.45 | 0.66 |
| 1. Wash up | 0.62 | 0.66 |
| 1. Put crockery/pans etc. into kitchen cupboards | 0.56 | 0.50 |
| 1. Use a kettle (e.g. fill, pour) | 0.53 | 0.67 |
| 1. Turn cooker knobs | 0.51 | 0.68 |
| 1. Open fridge door | 0.68 | 0.73 |
| 1. Prepare and cook snack and/or meal | 0.61 | 0.82 |

| **Domain 6: Moving around Indoors** | Dutch  n = 252 | German  n=163 |
| --- | --- | --- |
| 1. Walk indoors (e.g. get to toilet/ bathroom; round kitchen) | 0.71 | 0.63 |
| 1. Open the front or back door | 0.47 | 0.45 |
| 1. Lock and unlock doors | 0.54 | 0.57 |
| 1. Get to the front door in time to answer | 0.67 | 0.58 |
| 1. Get to the phone in time to answer | 0.50 | 0.61 |
| 1. Stand for longer periods | 0.65 | 0.68 |
| 1. Get up and down steps/ stairs | 0.55 | 0.74 |
| 1. Bend to floor/pick up items | 0.61 | 0.72 |
| 1. Reach up | 0.64 | 0.67 |
| 1. Kneel | 0.74 | 0.79 |
| 1. Carry heavy items around the house | 0.62 | 0.67 |
| 1. Manage heating (e.g. controls, woodburner, multifuel stove, open fire) | 0.40 | 0.55 |

| **Domain 7: Cleaning the House** | Dutch  n = 252 | German  n=163 |
| --- | --- | --- |
| 1. Make the bed | 0.71 | 0.63 |
| 1. Dust and wipe surfaces | 0.72 | 0.61 |
| 1. Sweep up/ mop floor | 0.59 | 0.63 |
| 1. Wring out a cloth | 0.64 | 0.64 |
| 1. Vacuum clean | 0.77 | 0.74 |
| 1. Open a window | 0.59 | 0.74 |
| 1. Clean windows | 0.64 | 0.72 |
| 1. Clean the bath and/or shower | 0.68 | 0.77 |
| 1. Heavy housework (e.g. move furniture, take down curtains) | 0.67 | 0.71 |

| **Domain 8: Laundry and Clothes Care** | Dutch  n = 252 | German  n=163 |
| --- | --- | --- |
| 1. Do the hand washing | 0.50 | 0.58 |
| 1. Use a washing machine (e.g. load and unload) | 0.73 | 0.58 |
| 1. Hang out washing | 0.73 | 0.68 |
| 1. Plug in and pull out an electric plug (any in home) | 0.48 | 0.63 |
| 1. Put up an ironing board | 0.60 | 0.64 |
| 1. Iron | 0.59 | 0.67 |
| 1. Do small repairs e.g. hemming, buttons | 0.70 | 0.67 |
| 1. Use scissors (any in home) | 0.51 | 0.62 |
| 1. Pick up pins/needles | 0.60 | 0.73 |

| **Domain 9: Moving and Transfers** | Dutch  n = 252 | German  n=163 |
| --- | --- | --- |
| 1. Get into and out of bed | 0.66 | 0.51 |
| 1. Turn over and sit up in bed | 0.65 | 0.50 |
| 1. Stand up from a chair without armrests | 0.62 | 0.64 |
| 1. Pull up bedclothes/duvet | 0.60 | 0.70 |
| 1. Getting a comfortable sleeping position | 0.62 | 0.53 |
| 1. Sit for longer periods (e.g. in a car, train) | 0.58 | 0.61 |

| **Domain 10: Communication** | Dutch  n = 252 | German  n=163 |
| --- | --- | --- |
| 1. Use a phone / mobile/ smartphone *(call/ text/ any functions)* | 0.61 | 0.53 |
| 1. Hold a book | 0.61 | 0.67 |
| 1. Write | 0.59 | 0.74 |
| 1. Handle money/ cards; use cash machine/ pay by card | 0.68 | 0.66 |
| 1. Use a computer and mouse/ laptop/ tablet (e.g. iPad) | 0.59 | 0.56 |
| 1. Use remote controls (e.g. TV) | 0.58 | 0.55 |

| **Domain 11:**  **Moving Outdoors and Shopping** | Dutch  n = 252 | German  n=163 |
| --- | --- | --- |
| 1. Walk on level ground | 0.60 | 0.41 |
| 1. Go for a long walk (e.g. a mile) | 0.70 | 0.68 |
| 1. Go up stairs without a handrail | 0.68 | 0.71 |
| 1. Travel by public transport | 0.70 | 0.59 |
| 1. Get in and out of a car and open car door | 0.66 | 0.57 |
| 1. Drive a car (e.g. hold steering wheel, turn car key, change gear) | 0.50 | 0.56 |
| 1. Fill the car with petrol | 0.52 | 0.67 |
| 1. Open a heavy (e.g. shop) door | 0.64 | 0.74 |
| 1. Walk around the shops | 0.53 | 0.78 |
| 1. Carry shopping | 0.57 | 0.74 |
| 1. Do the weekly shopping | 0.61 | 0.65 |
| 1. Hold a walking stick | 0.41 | 0.53 |
| 1. Use a mobility scooter | 0.33 | 0.53 |

| **Domain 12:**  **Gardening and Household Maintenance** | Dutch  n = 252 | German  n=163 |
| --- | --- | --- |
| 1. Change a light bulb | 0.53 | 0.63 |
| 1. Light gardening (e.g. weed, prune, plant) | 0.49 | 0.68 |
| 1. Heavy gardening (e.g. dig, mow) | 0.52 | 0.73 |
| 1. Climb ladders | 0.41 | 0.75 |
| 1. Clean the car (inside and out) | 0.45 | 0.80 |
| 1. Do household repairs | 0.46 | 0.78 |
| 1. Car maintenance (eg oil, water) | 0.46 | 0.78 |

| **Domain 13: Caring** | Dutch  n = 252 | German  n=163 |
| --- | --- | --- |
| 1. Feed a child, prepare bottles | 0.59 | 0.49 |
| 1. Bathe a child/ change nappy | 0.43 | 0.64 |
| 1. Dress a child | 0.34 | 0.72 |
| 1. Do a child’s hair | 0.33 | 0.55 |
| 1. Use children’s equipment (e.g. highchair, push chair, car seat) | 0.43 | 0.64 |
| 1. Put a child in/ out of highchair, push chair, high seat | 0.60 | 0.67 |
| 1. Lift and carry a child | 0.55 | 0.61 |
| 1. Play with children | 0.40 | 0.56 |
| 1. Care for others (e.g. elderly relatives) | 0.20 | 0.47 |

| **Domain 14. Leisure, Hobbies and Social Activities** | Dutch  n = 252 | German  n=163 |
| --- | --- | --- |
| 1. Crafts (e.g. knitting, crochet, sewing, embroidery, model making) | 0.62 | 0.68 |
| 1. Do-It-Yourself (e.g. using tools, painting and decorating) | 0.56 | 0.58 |
| 1. Visit friends/ socializing (e.g. pub, cinema, theatre) | 0.63 | 0.62 |
| 1. Attend community / religious groups or classes | 0.55 | 0.59 |
| 1. Physical activities (e.g. dance, active sports, swimming, bicycling) | 0.45 | 0.65 |
| 1. Quiet recreation (e.g. painting, cards) | 0.65 | 0.63 |
| 1. Performing arts (e.g. music, choir, dramatics) | 0.36 | 0.62 |
| 1. Pet care (e.g. feed, groom) | 0.45 | 0.70 |
| 1. Take dog for a walk (e.g. hold leash) | 0.25 | 0.67 |

**Supplementary Table S3.** Rasch Transformation: Evaluation of Daily Activity Questionnaire Self-Care Component for Dutch, German and English versions for Rheumatoid Arthritis.

| **Raw Score**  **(bold)** | **Rasch Transformed**  **Score** |
| --- | --- |
| **0** | 0.0 |
| **1** | 20.0 |
| **2** | 32.3 |
| **3** | 40.0 |
| **4** | 45.5 |
| **5** | 49.8 |
| **6** | 53.2 |
| **7** | 56.1 |
| **8** | 58.6 |
| **9** | 60.8 |
| **10** | 62.7 |
| **11** | 64.5 |
| **12** | 66.0 |
| **13** | 67.5 |
| **14** | 68.8 |
| **15** | 70.0 |
| **16** | 71.2 |
| **17** | 72.3 |
| **18** | 73.3 |
| **19** | 74.2 |
| **20** | 75.2 |
| **21** | 76.0 |
| **22** | 76.9 |
| **23** | 77.6 |
| **24** | 78.4 |
| **25** | 79.1 |
| **26** | 79.9 |
| **27** | 80.5 |
| **28** | 81.2 |
| **29** | 81.8 |
| **30** | 82.5 |
| **31** | 83.0 |
| **32** | 83.6 |
| **33** | 84.2 |
| **34** | 84.8 |
| **35** | 85.3 |
| **36** | 85.9 |
| **37** | 86.4 |
| **38** | 86.9 |
| **39** | 87.4 |
| **40** | 87.8 |
| **41** | 88.3 |
| **42** | 88.8 |
| **43** | 89.3 |
| **44** | 89.7 |
| **45** | 90.2 |
| **46** | 90.6 |
| **47** | 91.0 |
| **48** | 91.5 |
| **49** | 91.9 |
| **50** | 92.3 |
| **51** | 92.7 |
| **52** | 93.1 |
| **53** | 93.5 |
| **54** | 93.9 |
| **55** | 94.3 |
| **56** | 94.7 |
| **57** | 95.1 |
| **58** | 95.5 |
| **59** | 95.8 |
| **60** | 96.2 |
| **61** | 96.6 |
| **62** | 96.9 |
| **63** | 97.3 |
| **64** | 97.6 |
| **65** | 98.0 |
| **66** | 98.3 |
| **67** | 98.7 |
| **68** | 99.0 |
| **69** | 99.4 |
| **70** | 99.7 |
| **71** | 100.0 |
| **72** | 100.4 |
| **73** | 100.7 |
| **74** | 101.1 |
| **75** | 101.4 |
| **76** | 101.7 |
| **77** | 102.0 |
| **78** | 102.3 |
| **79** | 102.7 |
| **80** | 103.0 |
| **81** | 103.3 |
| **82** | 103.6 |
| **83** | 103.9 |
| **84** | 104.2 |
| **85** | 104.6 |
| **86** | 104.9 |
| **87** | 105.2 |
| **88** | 105.4 |
| **89** | 105.8 |
| **90** | 106.1 |
| **91** | 106.4 |
| **92** | 106.7 |
| **93** | 107.0 |
| **94** | 107.2 |
| **95** | 107.5 |
| **96** | 107.8 |
| **97** | 108.1 |
| **98** | 108.4 |
| **99** | 108.7 |
| **100** | 109.0 |
| **101** | 109.3 |
| **102** | 109.6 |
| **103** | 109.9 |
| **104** | 110.2 |
| **105** | 110.4 |
| **106** | 110.7 |
| **107** | 111.0 |
| **108** | 111.3 |
| **109** | 111.6 |
| **110** | 111.8 |
| **111** | 112.1 |
| **112** | 112.4 |
| **113** | 112.7 |
| **114** | 113.0 |
| **115** | 113.2 |
| **116** | 113.5 |
| **117** | 113.8 |
| **118** | 114.1 |
| **119** | 114.3 |
| **120** | 114.6 |
| **121** | 114.9 |
| **122** | 115.2 |
| **123** | 115.4 |
| **124** | 115.7 |
| **125** | 116.0 |
| **126** | 116.2 |
| **127** | 116.5 |
| **128** | 116.8 |
| **129** | 117.1 |
| **130** | 117.4 |
| **131** | 117.6 |
| **132** | 117.9 |
| **133** | 118.2 |
| **134** | 118.4 |
| **135** | 118.7 |
| **136** | 119.0 |
| **137** | 119.3 |
| **138** | 119.5 |
| **139** | 119.8 |
| **140** | 120.1 |
| **141** | 120.4 |
| **142** | 120.6 |
| **143** | 120.9 |
| **144** | 121.2 |
| **145** | 121.5 |
| **146** | 121.7 |
| **147** | 122.0 |
| **148** | 122.3 |
| **149** | 122.6 |
| **150** | 122.9 |
| **151** | 123.1 |
| **152** | 123.4 |
| **153** | 123.7 |
| **154** | 124.0 |
| **155** | 124.3 |
| **156** | 124.6 |
| **157** | 124.9 |
| **158** | 125.2 |
| **159** | 125.5 |
| **160** | 125.8 |
| **161** | 126.1 |
| **162** | 126.4 |
| **163** | 126.7 |
| **164** | 127.1 |
| **165** | 127.4 |
| **166** | 127.8 |
| **167** | 128.1 |
| **168** | 128.4 |
| **169** | 128.8 |
| **170** | 129.1 |
| **171** | 129.5 |
| **172** | 129.9 |
| **173** | 130.3 |
| **174** | 130.7 |
| **175** | 131.0 |
| **176** | 131.5 |
| **177** | 131.9 |
| **178** | 132.3 |
| **179** | 132.8 |
| **180** | 133.3 |
| **181** | 133.7 |
| **182** | 134.2 |
| **183** | 134.7 |
| **184** | 135.3 |
| **185** | 135.8 |
| **186** | 136.4 |
| **187** | 137.0 |
| **188** | 137.7 |
| **189** | 138.3 |
| **190** | 139.0 |
| **191** | 139.8 |
| **192** | 140.6 |
| **193** | 141.4 |
| **194** | 142.3 |
| **195** | 143.2 |
| **196** | 144.2 |
| **197** | 145.3 |
| **198** | 146.4 |
| **199** | 147.6 |
| **200** | 148.9 |
| **201** | 150.3 |
| **202** | 151.8 |
| **203** | 153.5 |
| **204** | 155.3 |
| **205** | 157.3 |
| **206** | 159.6 |
| **207** | 162.2 |
| **208** | 165.4 |
| **209** | 169.3 |
| **210** | 174.3 |
| **211** | 181.5 |
| **212** | 193.4 |
| **213** | 213.0 |

**Supplementary Table S4.** Rasch Transformation Evaluation of Daily Activity Questionnaire Mobility component for Dutch, German and English versions for Rheumatoid Arthritis.

| **Raw Score**  **(bold)** | **Rasch Transformed Score** | **24** | 67.4 | **49** | 88.5 | **74** | 98.1 | **99** | 102.5 |
| --- | --- | --- | --- | --- | --- | --- | --- | --- | --- |
| **0** | 0.0 | **25** | 68.5 | **50** | 89.0 | **75** | 98.3 | **100** | 102.7 |
| **1** | 14.8 | **26** | 69.6 | **51** | 89.6 | **76** | 98.6 | **101** | 102.8 |
| **2** | 23.9 | **27** | 70.7 | **52** | 90.1 | **77** | 98.8 | **102** | 102.9 |
| **3** | 29.5 | **28** | 71.8 | **53** | 90.6 | **78** | 99.0 | **103** | 103.1 |
| **4** | 33.6 | **29** | 72.8 | **54** | 91.1 | **79** | 99.2 | **104** | 103.2 |
| **5** | 36.9 | **30** | 73.8 | **55** | 91.6 | **80** | 99.4 | **105** | 103.4 |
| **6** | 39.6 | **31** | 74.8 | **56** | 92.0 | **81** | 99.6 | **106** | 103.6 |
| **7** | 42.0 | **32** | 75.8 | **57** | 92.5 | **82** | 99.8 | **107** | 103.7 |
| **8** | 44.1 | **33** | 76.7 | **58** | 92.9 | **83** | 99.9 | **108** | 103.9 |
| **9** | 46.1 | **34** | 77.6 | **59** | 93.3 | **84** | 100.1 | **109** | 104.1 |
| **10** | 47.8 | **35** | 78.5 | **60** | 93.7 | **85** | 100.3 | **110** | 104.2 |
| **11** | 49.6 | **36** | 79.4 | **61** | 94.1 | **86** | 100.5 | **111** | 104.4 |
| **12** | 51.2 | **37** | 80.2 | **62** | 94.5 | **87** | 100.7 | **112** | 104.6 |
| **13** | 52.7 | **38** | 81.0 | **63** | 94.8 | **88** | 100.8 | **113** | 104.8 |
| **14** | 54.3 | **39** | 81.8 | **64** | 95.2 | **89** | 101.0 | **114** | 104.9 |
| **15** | 55.7 | **40** | 82.6 | **65** | 95.5 | **90** | 101.1 | **115** | 105.2 |
| **16** | 57.1 | **41** | 83.3 | **66** | 95.8 | **91** | 101.3 | **116** | 105.4 |
| **17** | 58.5 | **42** | 84.0 | **67** | 96.2 | **92** | 101.5 | **117** | 105.6 |
| **18** | 59.9 | **43** | 84.7 | **68** | 96.5 | **93** | 101.6 | **118** | 105.8 |
| **19** | 61.2 | **44** | 85.4 | **69** | 96.8 | **94** | 101.7 | **119** | 106.0 |
| **20** | 62.5 | **45** | 86.0 | **70** | 97.0 | **95** | 101.9 | **120** | 106.3 |
| **21** | 63.9 | **46** | 86.7 | **71** | 97.3 | **96** | 102.1 | **121** | 106.5 |
| **22** | 65.2 | **47** | 87.3 | **72** | 97.6 | **97** | 102.2 | **122** | 106.7 |
| **23** | 66.4 | **48** | 87.9 | **73** | 97.8 | **98** | 102.3 | **123** | 107.0 |
| **124** | 107.3 |  |  |  |  |  |  |  |  |
| **125** | 107.6 |  |  |  |  |  |  |  |  |
| **126** | 107.9 |  |  |  |  |  |  |  |  |
| **127** | 108.3 |  |  |  |  |  |  |  |  |
| **128** | 108.6 |  |  |  |  |  |  |  |  |
| **129** | 109.0 |  |  |  |  |  |  |  |  |
| **130** | 109.4 |  |  |  |  |  |  |  |  |
| **131** | 109.8 |  |  |  |  |  |  |  |  |
| **132** | 110.3 |  |  |  |  |  |  |  |  |
| **133** | 110.8 |  |  |  |  |  |  |  |  |
| **134** | 111.4 |  |  |  |  |  |  |  |  |
| **135** | 112.0 |  |  |  |  |  |  |  |  |
| **136** | 112.7 |  |  |  |  |  |  |  |  |
| **137** | 113.4 |  |  |  |  |  |  |  |  |
| **138** | 114.2 |  |  |  |  |  |  |  |  |
| **139** | 115.1 |  |  |  |  |  |  |  |  |
| **140** | 116.2 |  |  |  |  |  |  |  |  |
| **141** | 117.5 |  |  |  |  |  |  |  |  |
| **142** | 119.0 |  |  |  |  |  |  |  |  |
| **143** | 121.0 |  |  |  |  |  |  |  |  |
| **144** | 123.6 |  |  |  |  |  |  |  |  |
| **145** | 127.5 |  |  |  |  |  |  |  |  |
| **146** | 134.4 |  |  |  |  |  |  |  |  |
| **147** | 147.0 |  |  |  |  |  |  |  |  |

**Supplementary Table S5: Evaluation of Daily Activity Questionnaire** **Part 2: discriminant validity by perceived health groups (Dutch n=239; German n=162): median (inter-quartile range) domain scores.**

| **EDAQ Part 2 domain section A (score range)** | **Good/Very Good** | **Fair** | **Poor/Very Poor** | **Chi-square** | **df** | **P <** |
| --- | --- | --- | --- | --- | --- | --- |
| **1: Eating and Drinking (0-33)** |  |  |  |  |  |  |
| Dutch | 3 (0-5) | 8 (4-11) | 13 (6.5-17) | 48.29 | 2 | 0.000 |
| German | 4 (0.5-7) | 8 (5-12) | 11.5 (8.5-16.5) | 40.37 | 2 | 0.000 |
| **2: In the Bathroom and Personal Care (0-36)** |  |  |  |  |  |  |
| Dutch | 0 (0-1) | 2 (0-5) | 7 (3.5-11) | 46.85 | 2 | 0.000 |
| German | 1 (0-3) | 4 (2-8) | 7.5 (1.25-10.5) | 36.75 | 2 | 0.000 |
| **3: Dressing (0-33)** |  |  |  |  |  |  |
| Dutch | 0.5 (0-3) | 5 (2-9) | 14 (7-18) | 58.18 | 2 | 0.000 |
| German | 2 (0-4) | 6.5 (3-13) | 8.5 (5-14) | 40.08 | 2 | 0.000 |
| **4: Bathing and Showering (0-33)** |  |  |  |  |  |  |
| Dutch | 1 (0-4) | 5 (1-10) | 11 (8-22) | 52.84 | 2 | 0.000 |
| German | 2 (0-5) | 6 (3-10.75) | 11 (5.25-16.75) | 42.25 | 2 | 0.000 |
| **5: Cooking (0-42)** |  |  |  |  |  |  |
| Dutch | 1 (0-4) | 5 (2-11) | 12 (7.5-28.5) | 51.20 | 2 | 0.000 |
| German | 2 (0-7) | 10 (6-15) | 14 (8.25-24.75) | 49.08 | 2 | 0.000 |
| **6: Moving Indoors (0-36)** |  |  |  |  |  |  |
| Dutch | 3 (0-7) | 8.5 (5-13.75) | 15 (10.5-22) | 52.54 | 2 | 0.000 |
| German | 3 (1-7) | 9 (7-14) | 13 (9.25-18) | 60.78 | 2 | 0.000 |
| **7: Cleaning the House (0-27)** |  |  |  |  |  |  |
| Dutch | 2 (0-6.25) | 8 (3.25-14.5) | 18 (6.5-25) | 46.31 | 2 | 0.000 |
| German | 3 (0-8) | 9 (6-14) | 14.5 (10.25-18) | 46.17 | 2 | 0.000 |
| **8: Laundry and Clothes Care (0-27)** |  |  |  |  |  |  |
| Dutch | 0 (0-2) | 4 (0-7.5) | 7 (2-19) | 38.77 | 2 | 0.000 |
| German | 2 (0-6) | 7 (3-12.5) | 11.5 (5.25-18.5) | 37.51 | 2 | 0.000 |
| **9: Moving and Transfers (0-18)** |  |  |  |  |  |  |
| Dutch | 1 (0-3) | 3 (2-6) | 10 (6-11.75) | 64.74 | 2 | 0.000 |
| German | 1 (0-3) | 5 (3.25-7) | 7.5 (4-9) | 62.37 | 2 | 0.000 |
| **10: Communication (0-18)** |  |  |  |  |  |  |
| Dutch | 0 (0-1) | 2 (1-3.75) | 3 (2-6) | 42.23 | 2 | 0.000 |
| German | 0 (0-2) | 3 (1-5) | 6 (2.25-7) | 38.18 | 2 | 0.000 |
| **11: Moving Outdoors and Shopping (0-39)** |  |  |  |  |  |  |
| Dutch | 3 (1-7) | 9.5 (5 – 13.25) | 20 (11.75-26) | 60.17 | 2 | 0.000 |
| German | 2.5 (0-7) | 10.5 (6-15.5) | 14.5 (11-21.5) | 61.24 | 2 | 0.000 |
| **12: Gardening and Household Maintenance (0-21)** |  |  |  |  |  |  |
| Dutch | 0 (1-4) | 3 (0.75-7) | 5 (1-9) | 12.27 | 2 | 0.002 |
| German | 2 (0-5) | 7.5 (5-12) | 13 (6.25-18) | 44.19 | 2 | 0.000 |
| **13: Caring (0-27)** |  |  |  |  |  |  |
| Dutch | 0 (0-0) | 0 (0-1) | 0 (0-1.5) | 2.61 | 2 | 0.27 |
| German | 0 (0-2) | 0.5 (0-7.75) | 3 (0-12.25) | 9.79 | 2 | 0.007 |
| **14: Leisure, Hobbies and Social Activities (0-27)** |  |  |  |  |  |  |
| Dutch | 1 (0-2) | 3 (1-5.5) | 7 (3.25-12.5) | 57.70 | 2 | 0.000 |
| German | 1 (0-3) | 5.5 (3-9) | 8.5 (7-13.5) | 52.16 | 2 | 0.000 |

Note: sample sizes Dutch: good/very good n =144; fair n = 79; poor/very poor n=16. German: very good/good n = 82; fair n = 60; poor/very poor n=20.
